# Supplementary material for: A combination of four Toxoplasma gondii nuclear-targeted effectors protects against interferon gamma-driven human host cell death
Source: mBio. 2024 Sep 18;15(10):e02124-24. doi: 10.1128/mbio.02124-24 (PMC11481881; doi:10.1128/mbio.02124-24)
Supplement: Supplemental Figures and Movie Legends — Figures S1-S8 and legends for supplemental movies. [file mbio.02124-24-s0001.pdf]

Figure S1

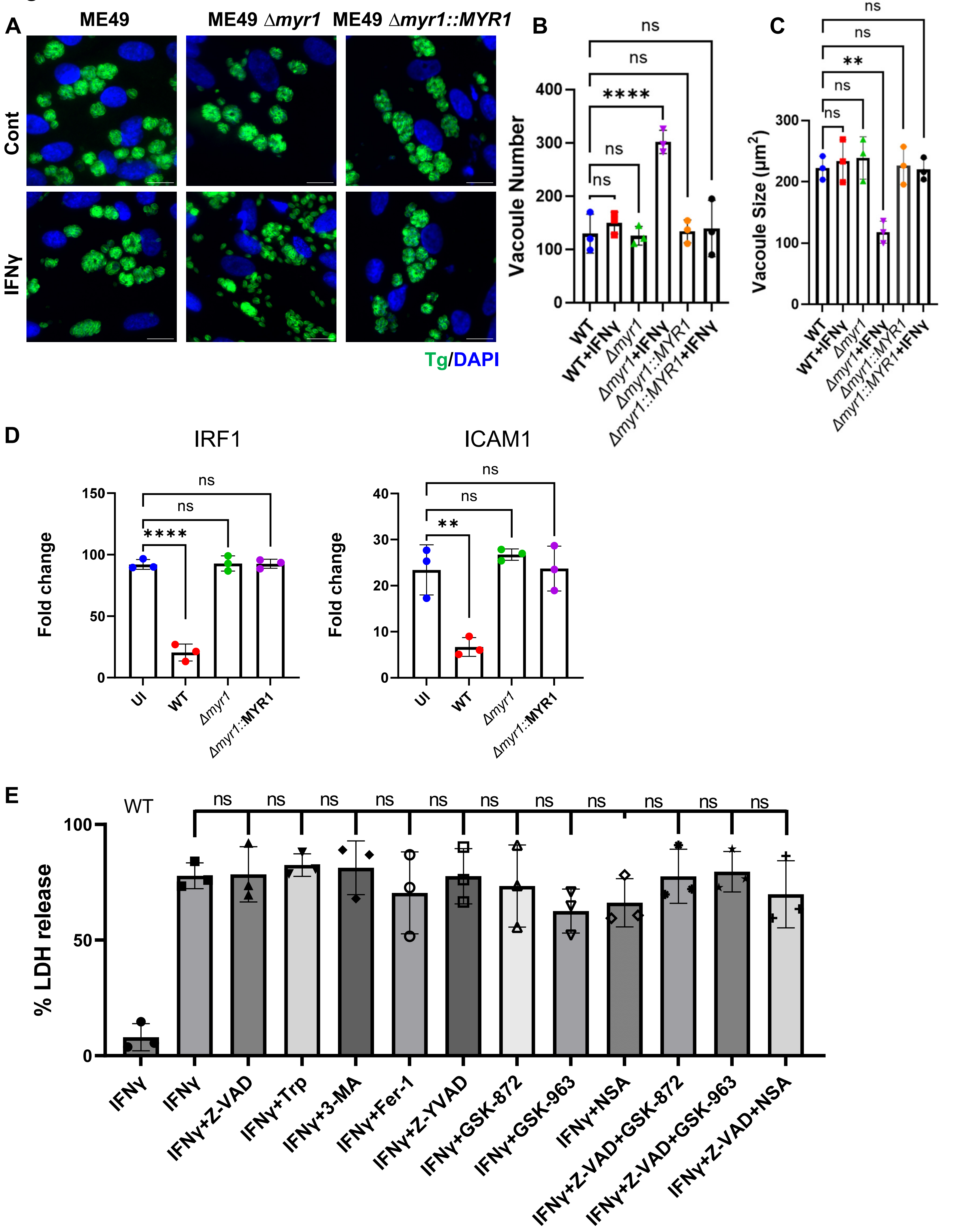

A
ME49
ME49 Δmyr1
ME49 Δmyr1::MYR1
Cont
IFNγ
Tg/DAPI
B
Vacoule Number
ns
ns
****
ns
ns
WT
WT+IFNγ
Δmyr1
Δmyr1+IFNγ
Δmyr1::MYR1
Δmyr1::MYR1+IFNγ
C
Vacoule Size (μm²)
ns
ns
**
ns
ns
WT
WT+IFNγ
Δmyr1
Δmyr1+IFNγ
Δmyr1::MYR1
Δmyr1::MYR1+IFNγ
D
IRF1
ICAM1
Fold change
ns
ns
****
**
UI
WT
Δmyr1
Δmyr1::MYR1
E
WT
% LDH release
ns
IFNγ
IFNγ+Z-VAD
IFNγ+Trp
IFNγ+3-MA
IFNγ+Fer-1
IFNγ+Z-YVAD
IFNγ+GSK-872
IFNγ+GSK-963
IFNγ+NSA
IFNγ+Z-VAD+GSK-872
IFNγ+Z-VAD+GSK-963
IFNγ+Z-VAD+NSA

Figure S1. MYR1 prevents early tachyzoite egress and host cell death in type II parasites.

(A) Representative images of HFF cells infected with ME49 (WT), ME49 Δ*myr1* and ME49 Δ*myr1*::MYR1 complement parasites for 24 hr prior to ± IFNγ 100 U/ml treatment. Forty-eight hours post infection cells were fixed and labelled with DAPI for nuclei (blue) and anti-GAP45 for parasites (green). Scale bar = 20 µm. (B) Average PV number per field. (C) Average PV size. Data in (B) and (C) represent Mean ± SD of three biological replicates conducted in technical duplicate with at least 30 images per sample and replicate. Statistical significance was determined using one-way ANOVA with Dunnett's multiple comparison test. ***P<0.001, ****P<0.0001.(D) qRT-PCR analysis of IFNγ induced transcripts of IRF1 and ICAM1 using RNA isolated from HFF cells infected with RH (WT), Δ*myr1*, or Δ*myr1::*MYR1 complemented parasites for 4 hr prior to IFNγ treatment (100 U/mL for 6 hr). Comparative cycle threshold (Ct) values were used to evaluate the fold change in transcripts using β−actin (ActB) as an internal transcript control. Data are plotted as fold change ± SEM from at least 3 independent experiments per gene. There were significant differences between the compared groups **P < 0.01, and ****P<0.0001 using one-way ANOVA with Tukey's multiple comparison test. (E) HFFs were infected with RH Δ*myr1* mutants for 4 hr before IFNγ 100 U/ml treatment in presence of cell death inhibitors Z-VAD-FMK (50 µM), GSK'963 (1 µM), GSK'872 (5 µM), NSA (10 µM), Z-YVAD-FMK (10 µM), 3-MA (5mM), Fer-1 (0.5 µM) and Tryptophan (Trp) (36 µg/ml). DMSO 0.05% only were used in control wells. Cell supernatant was collected 26 hr after infection and LDH activity was determined to measure cell lysis. Plotted is the percent of LDH release compared to maximal LDH release (after triton treatment of cells). Data from three independent experiments were pooled. Mean ± SD (n = 3 experiments, each with 3 technical replicates counted in each treatment) Statistical significance was determined using one-way ANOVA with Dunnett's multiple comparison test.

Figure S2

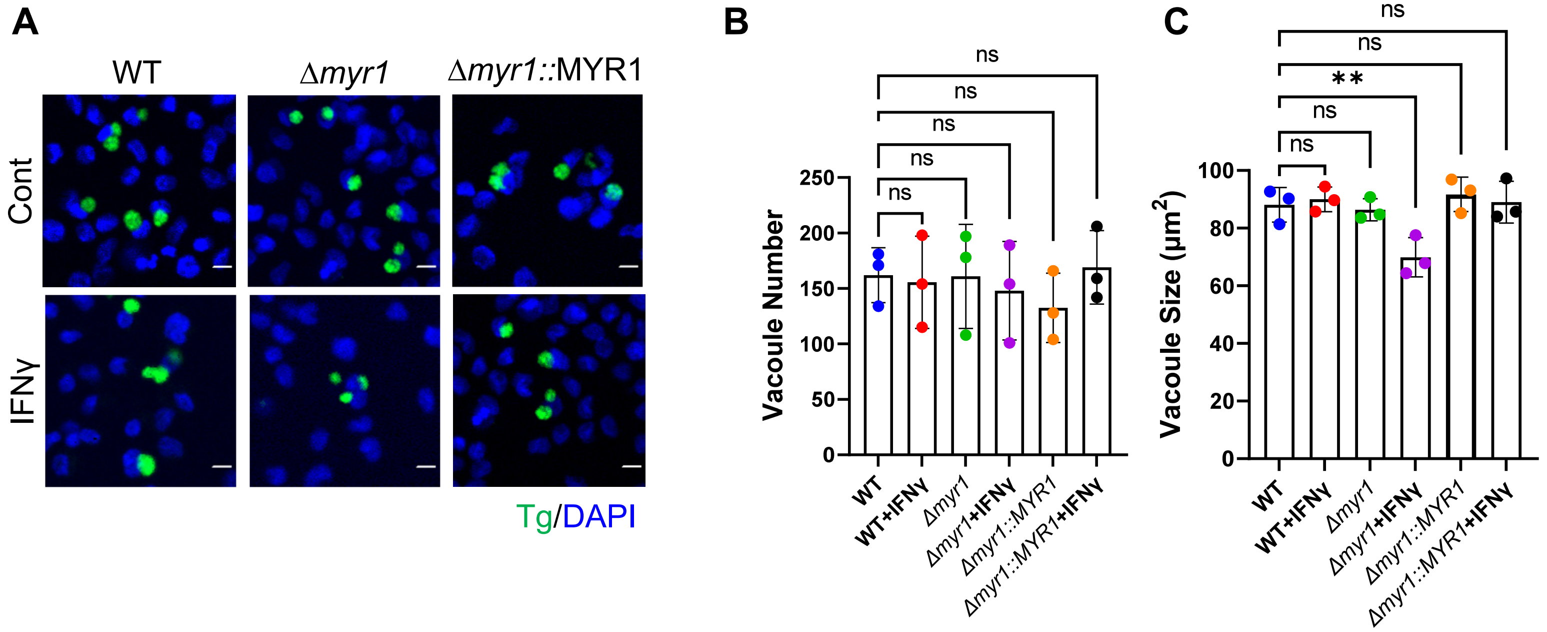
A
WT
Δmyr1
Δmyr1::MYR1
Cont
IFNγ
Tg/DAPI
B
ns
ns
ns
ns
ns
Vacoule Number
250
200
150
100
50
0
WT
WT+IFNγ
Δmyr1
Δmyr1+IFNγ
Δmyr1::MYR1
Δmyr1::MYR1+IFNγ
C
ns
ns
**
ns
ns
Vacoule Size (μm2)
100
80
60
40
20
0
WT
WT+IFNγ
Δmyr1
Δmyr1+IFNγ
Δmyr1::MYR1
Δmyr1::MYR1+IFNγ

Figure S2. Effect of MYR1 depletion on *T. gondii* response to IFNγ in human THP-1 macrophages.

(A) Representative images of THP-1 cells infected with RH (WT), Δ*myr1* and Δ*myr1*::MYR1 complement parasites for 4 hr prior to ± IFNγ 100 U/ml treatment. Twenty-six hours post infection cells were fixed and labelled with DAPI for nuclei (blue) and anti-GAP45 for parasites (green). Scale bar = 20 μm. (B) Average PV number per field. (C) Average PV size. Data in (B) and (C) represent Mean ± SD of three biological replicates conducted in technical duplicate with at least 30 images per sample and replicate. Statistical significance was determined using one-way ANOVA with Dunnett's multiple comparison test, $^{**}P<0.01$.

Figure S3

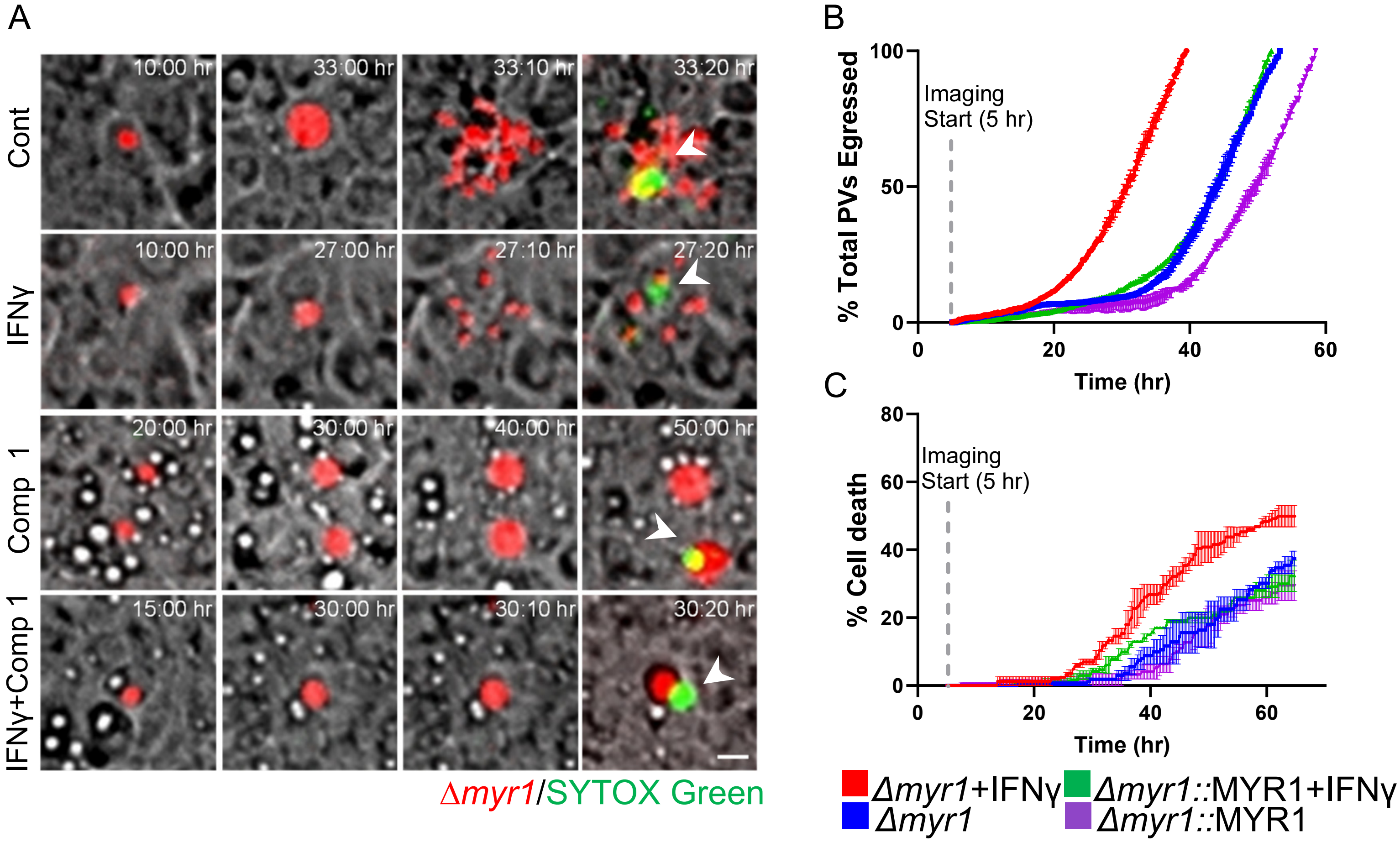
A
Cont
IFNγ
Comp 1
IFNγ+Comp 1
10:00 hr
33:00 hr
33:10 hr
33:20 hr
10:00 hr
27:00 hr
27:10 hr
27:20 hr
20:00 hr
30:00 hr
40:00 hr
50:00 hr
15:00 hr
30:00 hr
30:10 hr
30:20 hr
Δ*myr1*/SYTOX Green
B
% Total PVs Egressed
Imaging Start (5 hr)
Time (hr)
0
20
40
60
0
50
100
C
% Cell death
Imaging Start (5 hr)
Time (hr)
0
20
40
60
80
Δ*myr1*+IFNγ
Δ*myr1*::MYR1+IFNγ
Δ*myr1*
Δ*myr1*::MYR1

Figure S3. Effect of MYR1 depletion on *T. gondii* response to IFNγ in human A549 lung epithelial cells.

(A) Time-lapse images of A549 cells infected for 4 hr with ∆*myr1*-mCherry mutants (red) prior to ± IFNγ 100 U/ml treatment in the presence of SYTOX green (150 nM) (green) combined with ± 5 μM Compound 1. Live infection was imaged every 10 min starting 5 hr postinfection until 60 hr postinfection. Scale bar = 5 μm. (B) Time of ± IFNγ stimulated ∆*myr1*-mCherry and ∆*myr1::*MYR1-mCherry parasites egress was recorded for at least 100 PVs per condition per replicate. The percentage of total parasites egressed by the end of each hour is indicated. (C) Time of ± IFNγ stimulated WT-mCherry, ∆*myr1*-mCherry and ∆*myr1::*MYR1-mCherry infected cells death in presence of Compound 1 (5 μM) was recorded for at least 100 PVs per condition per replicate. Data from three independent experiments were pooled. The percentage of total infected host cells dead by the end of each hour is indicated.

Figure S4

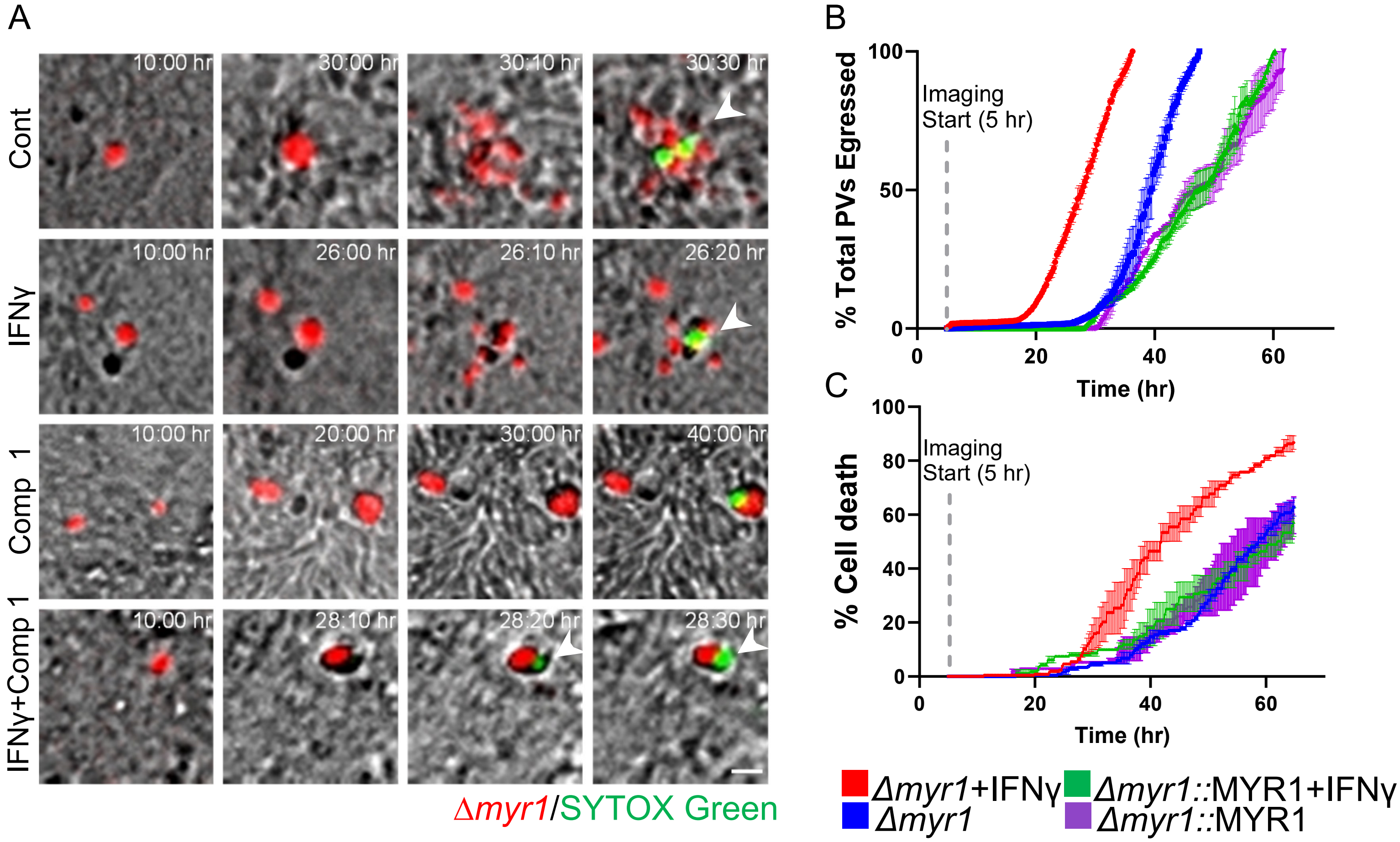
A
10:00 hr
30:00 hr
30:10 hr
30:30 hr
Cont
26:00 hr
26:10 hr
26:20 hr
IFNγ
20:00 hr
30:00 hr
40:00 hr
Comp 1
28:10 hr
28:20 hr
28:30 hr
IFNγ+Comp 1
Δmyr1/SYTOX Green
B
% Total PVs Egressed
Imaging Start (5 hr)
Time (hr)
C
% Cell death
Imaging Start (5 hr)
Time (hr)
Δmyr1+IFNγ
Δmyr1::MYR1+IFNγ
Δmyr1
Δmyr1::MYR1

Figure S4. Effect of MYR1 depletion on *T. gondii* response to IFNγ in human HCT-8 colon epithelial cells.

(A) Time-lapse images of HCT-8 cells infected for 4 hr with Δ*myr1*-mCherry mutants (red) prior to ± IFNγ 100 U/ml treatment in the presence of SYTOX green (150 nM) (green) combined with ± 5 μM Compound 1. Live infection was imaged every 10 min starting 5 hr postinfection until 60 hr postinfection. Scale bar = 5 μm. (B) Time of ± IFNγ stimulated Δ*myr1*-mCherry and Δ*myr1::*MYR1-mCherry parasites egress was recorded for at least 100 PVs per condition per replicate. The percentage of total parasites egressed by the end of each hour is indicated. (C) Time of ± IFNγ stimulated WT-mCherry, Δ*myr1*-mCherry and Δ*myr1::*MYR1-mCherry infected cells death in presence of Compound 1 (5 μM) was recorded for at least 100 PVs per condition per replicate. Data from three independent experiments were pooled. The percentage of total infected host cells dead by the end of each hour is indicated.

Figure S5

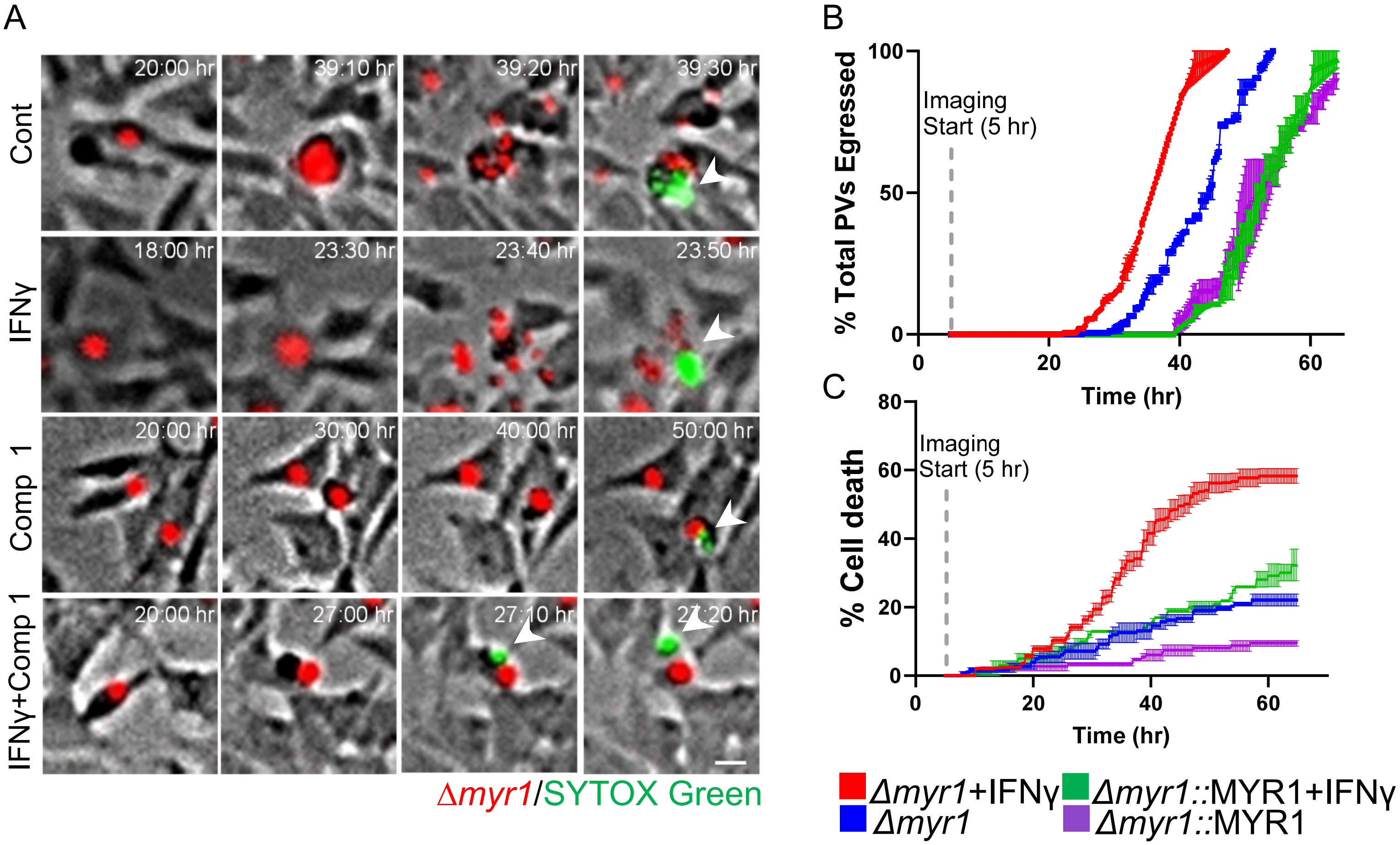
A
Cont
IFNγ
Comp 1
IFNγ+Comp 1
20:00 hr
39:10 hr
39:20 hr
39:30 hr
18:00 hr
23:30 hr
23:40 hr
23:50 hr
20:00 hr
30:00 hr
40:00 hr
50:00 hr
20:00 hr
27:00 hr
27:10 hr
27:20 hr
Δmyr1/SYTOX Green
B
% Total PVs Egressed
Imaging Start (5 hr)
Time (hr)
C
% Cell death
Imaging Start (5 hr)
Time (hr)
Δmyr1+IFNγ
Δmyr1::MYR1+IFNγ
Δmyr1
Δmyr1::MYR1

Figure S5. Effect of MYR1 depletion on *T. gondii* response to IFNγ in human SH-SY5Y neuroblastoma cells.

(A) Time-lapse images of SH-SY5Y cells infected for 4 hr with ∆*myr1*-mCherry mutants (red) prior to ± IFNγ 100 U/ml treatment in the presence of SYTOX green (150 nM) (green) combined with ± 5 µM Compound 1. Live infection was imaged every 10 min starting 5 hr postinfection until 60 hr postinfection. Scale bar = 5 µm. (B) Time of ± IFNγ stimulated ∆*myr1*-mCherry and ∆*myr1::*MYR1-mCherry parasites egress was recorded for at least 100 PVs per condition per replicate. The percentage of total parasites egressed by the end of each hour is indicated. (C) Time of ± IFNγ stimulated WT-mCherry, ∆*myr1*-mCherry and ∆*myr1::*MYR1-mCherry infected cells death in presence of Compound 1 (5 µM) was recorded for at least 100 PVs per condition per replicate. Data from three independent experiments were pooled. The percentage of total infected host cells dead by the end of each hour is indicated.

Figure S6

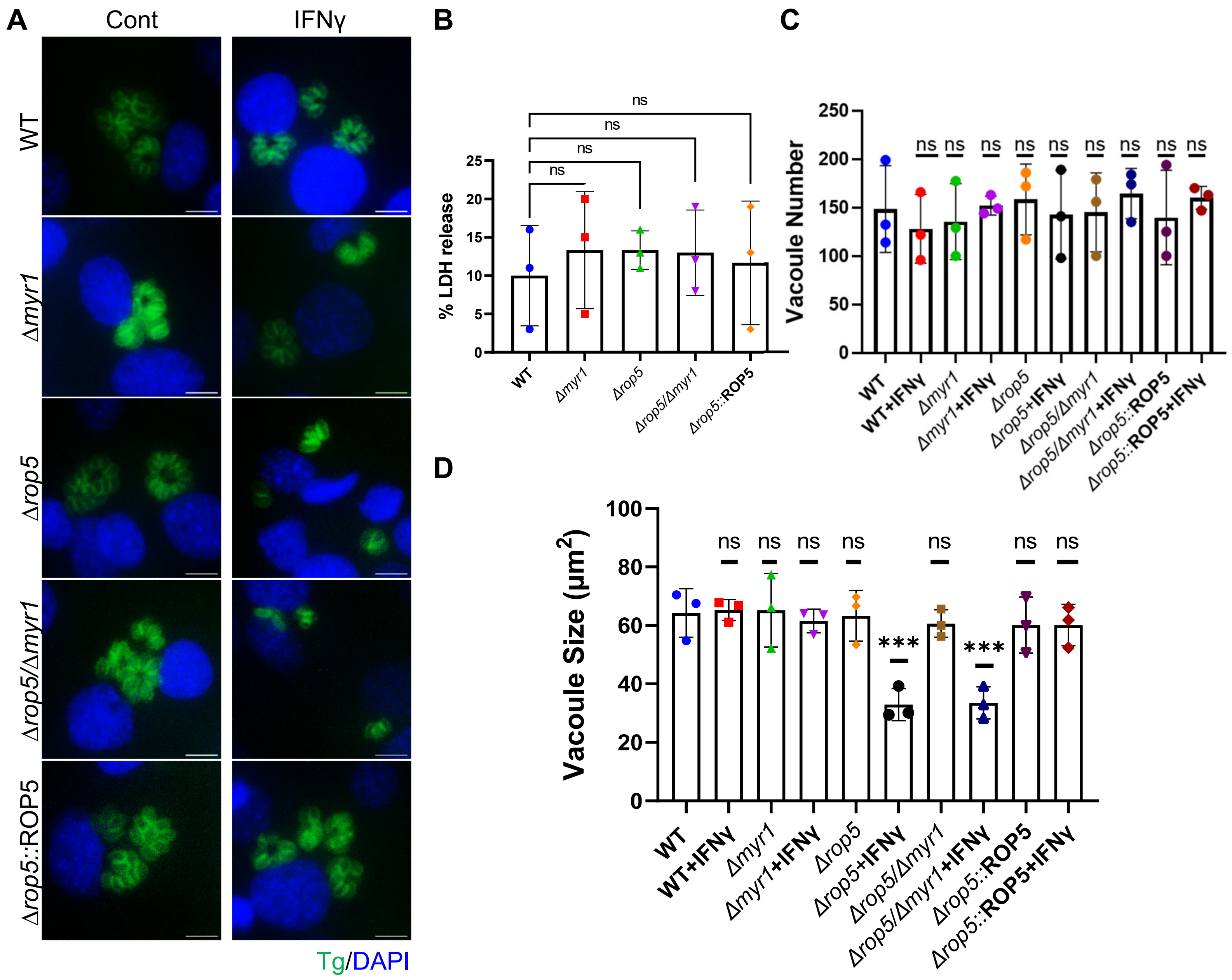
A
Cont
IFNγ
WT
Δmyr1
Δrop5
Δrop5/Δmyr1
Δrop5::ROP5
Tg/DAPI
B
% LDH release
ns
ns
ns
ns
WT
Δmyr1
Δrop5
Δrop5/Δmyr1
Δrop5::ROP5
C
Vacoule Number
WT
WT+IFNγ
Δmyr1
Δmyr1+IFNγ
Δrop5
Δrop5+IFNγ
Δrop5/Δmyr1
Δrop5/Δmyr1+IFNγ
Δrop5::ROP5
Δrop5::ROP5+IFNγ
D
Vacoule Size (μm2)
***
***
ns
WT
WT+IFNγ
Δmyr1
Δmyr1+IFNγ
Δrop5
Δrop5+IFNγ
Δrop5/Δmyr1
Δrop5/Δmyr1+IFNγ
Δrop5::ROP5
Δrop5::ROP5+IFNγ

Figure S6. Effect of ROP5 depletion on *T. gondii* response to IFNγ in murine fibroblasts.

(A) Representative images of L929 cells infected with RH (WT), Δ*myr1*, Δ*rop5*, Δ*rop5/*Δ*myr1* and Δ*rop5*::ROP5 complement parasites for 4 hr prior to ± IFNγ 100 U/ml treatment. 26 hr post infection cells were fixed and labelled with DAPI for nuclei (blue) and anti-GAP45 for parasites (green). Scale bar = 20 μm. (B) LDH release in the supernatant of L929 cells was measured 26 hr post infection. Plotted is the percent of LDH release compared to maximal LDH release (after triton treatment of cells). (C) Average PV number per field, (D) Average PV size. Data in (B) represents Mean ± SD of three biological replicates Mean ± SD (n = 3 experiments, each with 3 technical replicates counted in each treatment). Statistical significance was determined using one-way ANOVA test with Dunnett's multiple comparison test. Data in (C) and (D) represent means ± SD of three biological replicates conducted in technical duplicate with at least 30 images per sample and replicate. Statistical significance was determined using one-way ANOVA with Dunnett's multiple comparison test. ***P<0.001.

Figure S7

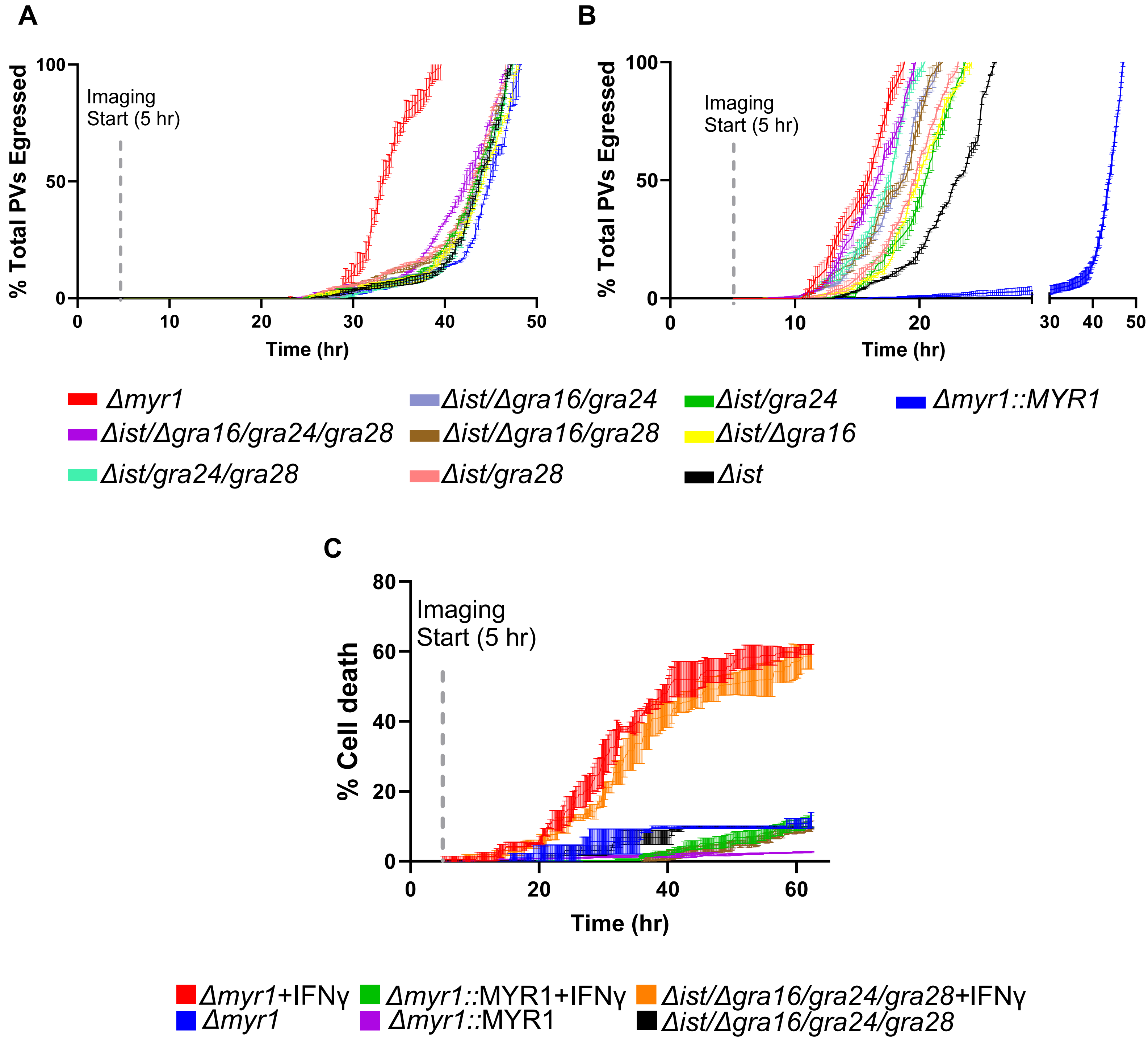
A
% Total PVs Egressed
100
50
0
Imaging
Start (5 hr)
0
10
20
30
40
50
Time (hr)
B
% Total PVs Egressed
100
50
0
Imaging
Start (5 hr)
0
10
20
30
40
50
Time (hr)
Δmyr1
Δist/Δgra16/gra24
Δist/gra24
Δmyr1::MYR1
Δist/Δgra16/gra24/gra28
Δist/Δgra16/gra28
Δist/Δgra16
Δist/gra24/gra28
Δist/gra28
Δist
C
% Cell death
80
60
40
20
0
Imaging
Start (5 hr)
0
20
40
60
Time (hr)
Δmyr1+IFNγ
Δmyr1::MYR1+IFNγ
Δist/Δgra16/gra24/gra28+IFNγ
Δmyr1
Δmyr1::MYR1
Δist/Δgra16/gra24/gra28

Figure S7. Effect of IST, GRA16, GRA24 and GRA28 depletion on *T. gondii* response to IFNγ in HFF cells.

(A), (B) Kinetics of parasite egress and host cell death. HFFs were infected with ∆*myr1*, ∆*myr1*::MYR1 complement, and different IFNγ-sensitive knockout combinations expressing mCherry: *Δist, Δist/Δgra16, Δist/Δgra24, Δist/Δgra28, ∆ist/∆gra16/∆gra24, ∆ist/∆gra16/∆gra28, ∆ist/∆gra24/∆gra28*, and *∆ist/∆gra16/∆gra24/∆gra28*. Cells were left untreated (A) or stimulated with IFNγ 100 U/ml 4 hours post-infection (B) and imaged every 10 minutes starting 5 hours post-infection until 60 hours post-infection. The time of parasite egress was recorded for at least 100 PVs per condition per replicate. The percentage of total parasites egressed by the end of each hour is indicated. Data from three independent experiments were pooled. (C) Time of ± IFNγ stimulated ∆*myr1*-mCherry, ∆*myr1::*MYR1-mCherry complement and ∆*ist/*∆*gra16/*∆*gra24/*∆*gra28*-mCherry infected HFFs death in presence of Compound 1 (5 μM) was recorded for at least 100 PVs per condition per replicate. Data from three independent experiments were pooled. The percentage of total infected host cells dead by the end of each hour is indicated.

Figure S8

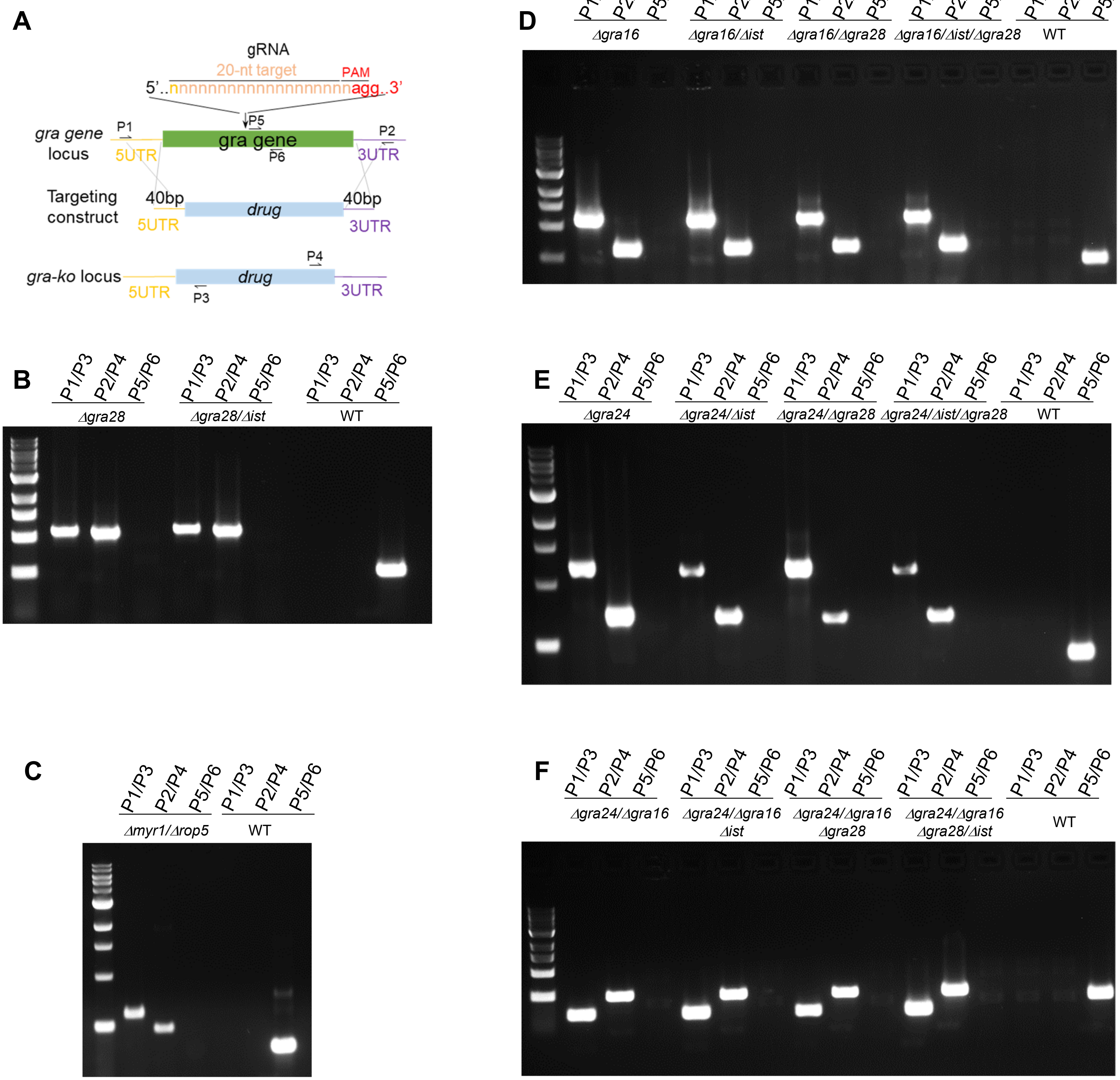
A
gRNA
20-nt target
PAM
5'..nnnnnnnnnnnnnnnnnnnnagg..3'
gra gene locus
P1
P5
gra gene
P6
P2
5UTR
3UTR
Targeting construct
40bp
drug
40bp
5UTR
3UTR
gra-ko locus
P4
drug
5UTR
P3
3UTR
B
P1/P3
P2/P4
P5/P6
Δgra28
Δgra28/Δist
WT
C
P1/P3
P2/P4
P5/P6
Δmyr1/Δrop5
WT
D
P1/P3
P2/P4
P5/P6
Δgra16
Δgra16/Δist
Δgra16/Δgra28
Δgra16/Δist/Δgra28
WT
E
P1/P3
P2/P4
P5/P6
Δgra24
Δgra24/Δist
Δgra24/Δgra28
Δgra24/Δist/Δgra28
WT
F
P1/P3
P2/P4
P5/P6
Δgra24/Δgra16
Δgra24/Δgra16 Δist
Δgra24/Δgra16 Δgra28
Δgra24/Δgra16 Δgra28/Δist
WT

Figure S8. Strategy for generation of knockout lines.

Schematic representation of the strategy for CRISPR/Cas9-mediated gene deletion used to generate transgenic strains used in this study. A single sgRNA expressing CRISPR/Cas9 (gRNA) plasmids targeting the middle of the genes were used to mediate double strand break and facilitate isolation of knockouts by homologous recombination. Targeting constructs consisted of a selection cassette (B) Δ*gra28*-hxgprt, (C) Δmyr1-dhfr, (D) Δ*gra16*-cat (E) *gra24*-cat and (F) *gra24*-phleo and short homology flanks (~40bp) immediately upstream of the translation initiation site (left arm) and downstream of the stop codon (right arm) as homologous arms to the flanking regions of the gene of interest. Diagnostic PCRs results (B-F) to verify locus disruption. The priming sites for PCR primers are indicated: P1/P3 and P2/4 confirm integration of left and right homologous arms, respectively; P5/6 examines the integrity of the endogenous gene. A successful knockout clone gave positive PCR products with P1/P3 and P2/4 but no product with P5/6, whereas the wild type parasites gave the opposite. See also Table S6 for oligonucleotide sequences.

Supplemental items

Movie S1. Live imaging of HFFs infected with RH Δ*myr1*-mCherry at 10 min intervals.

HFF cells were infected with RH Δ*myr1*-mCherry. Live infection was imaged every 10 min starting 5 hr postinfection for 43 hr.

Movie S2. Live imaging of HFFs infected with RH Δ*myr1*-mCherry at 10 min intervals in presence of IFNγ. HFF cells were infected with RH Δ*myr1*-mCherry and stimulated with IFNγ (100 u/ml) 4 hr post infection. Live infection was imaged every 10 min starting 5 hr postinfection for 43 hr.

Movie S3. Live imaging of HFFs infected with RH Δ*myr1*-mCherry at 10 min intervals in presence of Compound 1. HFF cells were infected with RH Δ*myr1*-mCherry and treated with Compound 1 (5µM) 4 hr post infection. Live infection was imaged every 10 min starting 5 hr postinfection for 58 hr.

Movie S4. Live imaging of HFFs infected with RH Δ*myr1*-mCherry in 10 min intervals in presence of Compound 1 and IFNγ. HFF cells were infected with RH Δ*myr1*-mCherry and treated with Compound 1 (5µM) and IFNγ (100 u/ml) 4 hr post infection. Live infection was imaged every 10 min starting 5 hr postinfection for 58 hr.
