# Supplementary material for: A combination of four Toxoplasma gondii nuclear-targeted effectors protects against interferon gamma-driven human host cell death
Source: mBio. 2024 Sep 18;15(10):e02124-24. doi: 10.1128/mbio.02124-24 (PMC11481881; doi:10.1128/mbio.02124-24)
Supplement: Table S4 — T. gondii lines used in this study. [file mbio.02124-24-s0004.docx]

**Table S4 – *Toxoplasma gondii* lines used in this study.**

| **Line** | **Genotype** | **Source** |
| --- | --- | --- |
| RH (WT) | RH*ΔhxgprtΔku80* | (Huynh and Carruthers, 2009) |
| RH (WT) mCherry | RH*ΔhxgprtΔku80, uprt::pGRA1:mCherry* | This study |
| RH *Δmyr1* | RH*Δhxgprt Δmyr1::HX,* mCherry | (Franco et al., 2016) |
| RH *Δmyr1/MYR1* | RH*Δhxgprt*  *Δmyr1/MYR1::HX, mCherry* | (Franco et al., 2016) |
| RH *Δrop5* | RH *ΔhxgprtΔku80 Δrop5::HX* | (Behnke et al., 2011) |
| RH *Δrop5/ROP5* | RH *ΔhxgprtΔku80 Δrop5/ROP5* | (Behnke et al., 2011) |
| RH *Δrop5, Δmyr1* | RH *ΔhxgprtΔku80 Δrop5::HX, Δmyr1:: DHFR-TS* | This study |
| RH cas9 | RH TUB1:cas9 P2ACAT | (Markus et al., 2019) |
| RH cas9 *Δgra28* | RH *Δhxgprt* TUB1:cas9:CAT1, *Δgra28::HX* | This study |
| RH GRA16-Ty | RH*ΔhxgprtΔku80*; GRA16-Ty, DHFR-TS | This study |
| RH GRA28-Ty | RH*ΔhxgprtΔku80*; GRA28-Ty, HX | This study |
| RH *Δist* | RH*ΔhxgprtΔku80*; *Δist::DHFR-TS* | (Kongsomboonvech et al., 2023) |
| RH *Δist/IST* | RH*ΔhxgprtΔku80*; *Δist::DHFR-TS/TgIST-Ty CAT* | (Kongsomboonvech et al., 2023) |
| RH *Δnsm* | RH*ΔhxgprtΔku80*; *Δnsm::HX* | (Kongsomboonvech et al., 2023) |
| RH *Δist,Δnsm* | RH*ΔhxgprtΔku80*; *Δist::DHFR-TS,* *Δnsm::HX* | (Kongsomboonvech et al., 2023) |
| RH *Δgra16* | RH*ΔhxgprtΔku80*; *Δgra16::CAT* | This study |
| RH *Δgra24* | RH*ΔhxgprtΔku80*; *Δgra24::CAT* | This study |
| RH *Δgra28* | RH*ΔhxgprtΔku80*; *Δgra28::HX* | This study |
| RH *Δist,Δgra16* | RH*ΔhxgprtΔku80*; *Δist::DHFR-TS, Δgra16::CAT* | This study |
| RH *Δist,Δgra16/GRA16* | RH*ΔhxgprtΔku80*; *Δist::DHFR-TS, Δgra16::CAT /uprt*::phleo,*GRA16-Ty* | This study |
| RH *Δist,Δgra24* | RH*ΔhxgprtΔku80*; *Δist::DHFR-TS, Δgra24::CAT* | This study |
| RH *Δist,Δgra24/GRA24* | RH*ΔhxgprtΔku80*; *Δist::DHFR-TS, Δgra24::CAT/uprt*::phleo,*GRA24-Ty* | This study |
| RH *Δist,Δgra28* | RH*ΔhxgprtΔku80*; *Δist::DHFR-TS, Δgra28::HX* | This study |
| RH *Δist,Δgra28/GRA28* | RH*ΔhxgprtΔku80*; *Δist::DHFR-TS, Δgra28::HX/uprt*::CAT,*GRA28-Ty* | This study |
| RH *Δgra16,Δgra24* | RH*ΔhxgprtΔku80*; *Δgra16::CAT, Δgra24::PHLEO* | This study |
| RH *Δgra16,Δgra28* | RH*ΔhxgprtΔku80*; *Δgra16::CAT, Δgra28::HX* | This study |
| RH *Δgra24,Δgra28* | RH*ΔhxgprtΔku80*; *Δgra24::CAT, Δgra28::HX* | This study |
| RH *Δist,Δgra16,Δgra24* | RH*ΔhxgprtΔku80*; *Δist::DHFR-TS, Δgra16::CAT, Δgra24::PHLEO* | This study |
| RH *Δist,Δgra16,Δgra28* | RH*ΔhxgprtΔku80*; *Δist::DHFR-TS, Δgra16::CAT, Δgra28::HX* | This study |
| RH *Δist,Δgra24,Δgra28* | RH*ΔhxgprtΔku80*; *Δist::DHFR-TS, Δgra24::CAT, Δgra28::HX* | This study |
| RH *Δgra16,Δgra24,Δgra28* | RH*ΔhxgprtΔku80*; *Δgra16::CAT, Δgra24::PHLEO , Δgra28::HX* | This study |
| RH *Δist,Δgra16,Δgra24,Δgra28* | RH*ΔhxgprtΔku80*; *Δist::DHFR-TS, Δgra16::CAT, Δgra24::PHLEO , Δgra28::HX* | This study |
| RH *Δist* | RH*ΔhxgprtΔku80*; *Δist::DHFR-TS, uprt::pGRA1:mCherry* | This study |
| RH *Δist,Δgra16* | RH*ΔhxgprtΔku80*; *Δist::DHFR-TS, Δgra16::CAT, uprt::pGRA1:mCherry* | This study |
| RH *Δist,Δgra24* | RH*ΔhxgprtΔku80*; *Δist::DHFR-TS, Δgra24::CAT, uprt::pGRA1:mCherry* | This study |
| RH *Δist,Δgra28* | RH*ΔhxgprtΔku80*; *Δist::DHFR-TS, Δgra28::HX, uprt::pGRA1:mCherry* | This study |
| RH *Δist,Δgra16,Δgra24* | RH*ΔhxgprtΔku80*; *Δist::DHFR-TS, Δgra16::CAT, Δgra24::PHLEO, uprt::pGRA1:mCherry* | This study |
| RH *Δist,Δgra16,Δgra28* | RH*ΔhxgprtΔku80*; *Δist::DHFR-TS, Δgra16::CAT, Δgra28::HX, uprt::pGRA1:mCherry* | This study |
| RH *Δist,Δgra24,Δgra28* | RH*ΔhxgprtΔku80*; *Δist::DHFR-TS, Δgra24::CAT, Δgra28::HX, uprt::pGRA1:mCherry* | This study |
| RH *Δist,Δgra16,Δgra24,Δgra28* | RH*ΔhxgprtΔku80*; *Δist::DHFR-TS, Δgra16::CAT, Δgra24::PHLEO , Δgra28::HX, uprt::pGRA1:mCherry* | This study |
| ME49 (WT) | ME49 *Δhxpgrt::FLUC* | (Tobin and Knoll, 2012) |
| ME49 *Δmyr1* | ME49 Δhxpgrt::FLUC; *Δmyr1::HX* | (Franco et al., 2016) |
| ME49 *Δmyr1/MYR1* | ME49 Δhxpgrt::FLUC; *Δmyr1/MYR1::HX* | (Franco et al., 2016) |

**References:**

Behnke, M.S., Khan, A., Wootton, J.C., Dubey, J.P., Tang, K., and Sibley, L.D. (2011). Virulence differences in Toxoplasma mediated by amplification of a family of polymorphic pseudokinases. Proc Natl Acad Sci U S A *108*, 9631-9636.

Franco, M., Panas, M.W., Marino, N.D., Lee, M.C., Buchholz, K.R., Kelly, F.D., Bednarski, J.J., Sleckman, B.P., Pourmand, N., and Boothroyd, J.C. (2016). A Novel Secreted Protein, MYR1, Is Central to Toxoplasma's Manipulation of Host Cells. mBio *7*, e02231-02215.

Huynh, M.H., and Carruthers, V.B. (2009). Tagging of endogenous genes in a Toxoplasma gondii strain lacking Ku80. Eukaryot Cell *8*, 530-539.

Kongsomboonvech, A.K., Garcia-Lopez, L., Njume, F., Rodriguez, F., Souza, S.P., Rosenberg, A., and Jensen, K.D.C. (2023). Variation in CD8 T cell IFNgamma differentiation to strains of Toxoplasma gondii is characterized by small effect QTLs with contribution from ROP16. Front Cell Infect Microbiol *13*, 1130965.

Markus, B.M., Bell, G.W., Lorenzi, H.A., and Lourido, S. (2019). Optimizing Systems for Cas9 Expression in Toxoplasma gondii. mSphere *4*.

Tobin, C.M., and Knoll, L.J. (2012). A patatin-like protein protects Toxoplasma gondii from degradation in a nitric oxide-dependent manner. Infect Immun *80*, 55-61.
