# Supplementary material for: A combination of four Toxoplasma gondii nuclear-targeted effectors protects against interferon gamma-driven human host cell death
Source: mBio. 2024 Sep 18;15(10):e02124-24. doi: 10.1128/mbio.02124-24 (PMC11481881; doi:10.1128/mbio.02124-24)
Supplement: Table S5 — Plasmids used in this study. [file mbio.02124-24-s0005.docx]

**Table S5. Plasmids used in this study.**

| **Common Name** | **Description** | **Source** |
| --- | --- | --- |
| pSAG1:CAS9-GFP, U6:sgUPRT | Template for making GOI targeting Cas9 plasmids | Addgene (#54467)  (Shen et al., 2014) |
| pBM019 | Construct for generating stable Cas9-expressing Toxoplasma gondii | Addgene (#128179)  (Markus et al., 2019) |
| pSAG1:CAS9-GFP, U6:sgUPRT | CRISPR plasmid targeting UPRT locus | (Shen et al., 2014) |
| pSAG1:CAS9-GFP, U6:sgGRA16 (3’) | CRISPR plasmid targeting GRA16 3’ UTR for C-terminal tagging | This study |
| pSAG1:CAS9-GFP, U6:sgGRA28 (3’) | CRISPR plasmid targeting GRA28 3’ UTR for C-terminal tagging | This study |
| pSAG1:CAS9-GFP, U6:sgGRA16 | CRISPR plasmid targeting GRA16 coding sequence for gene deletion | This study |
| pSAG1:CAS9-GFP, U6:sgGRA24 | CRISPR plasmid targeting GRA24 coding sequence for gene deletion | This study |
| pSAG1:CAS9-GFP, U6:sgGRA28 | CRISPR plasmid targeting GRA28 coding sequence for gene deletion | This study |
| pSAG1:CAS9-GFP, U6:sgMYR1 | CRISPR plasmid targeting MYR1 coding sequence for gene deletion | This study |
| pTgNSM-Ty-complement-CAT | Subcloning; for generating *GAR28-Ty, GRA16-Ty and GAR24-Ty* complement plasmids. | (Rosenberg and Sibley, 2021) |
| pFloxed DHFR-TS* | Template for amplification of DHFR resistance cassette for obtaining deletion strains | In-house |
| pLinker-2xTy-HXGPRT-LoxP | Template for amplification of HX resistance cassette for obtaining deletion strains and for obtaining endogenously TY tagged proteins | In-house |
| pLinker-2xTy-DHFR-LoxP | Template for amplification of DHFR resistance cassette for obtaining endogenously TY tagged proteins | In-house |
| pBS-SAG1-CAT | Template for amplification of CAT resistance cassette for obtaining pTgNSM-Ty-complement-CAT and pTgIST-Ty-complement-CAT plasmids | In-house |
| Tet-on-TAP-PHLEO | Template for amplification of PHLEO resistance cassette for obtaining pGRA16-Ty-complement-PHLEO ,pGRA16-Ty-complement-PHLEO plasmids and GRA24 knockout strains | (Etheridge et al., 2014) |
| pUPRT-5UTR-GRA16-Ty-3UTR-PHLEO | *GRA16-2Ty* fusion with *PHLEO* drug selectable marker flanked by homology arms from *UPRT*. Used with pSAG1:CAS9-GFP, U6:sgUPRT to obtain GRA16 complement strain | This study |
| pUPRT-5UTR-GRA24-TY-3UTR-PHLEO | *GRA24-2Ty* fusion with *PHLEO* drug selectable marker flanked by homology arms from *UPRT*. Used with pSAG1:CAS9-GFP, U6:sgUPRT to obtain GRA24 complement strain | This study |
| pUPRT-5UTR-GRA28-TY-3UTR-CAT | *GRA28-2Ty* fusion with *CAT* drug selectable marker flanked by homology arms from *UPRT*. Used with pSAG1:CAS9-GFP, U6:sgUPRT to obtain GRA28 complement strain | This study |
| pGRA1-mCherry-SAG1-DHFR | Template for amplification of pGRA1-mCherry cassette for obtaining mCherry expressing strains. | In-house |
| pSG5-HA-p300 | Full length HA tagged p300 protein expression vector | Addgene #89094  (Askew et al., 2010) |
| pRc/RSV-m CBP-HA | Full length HA tagged CBP protein expression vector | Addgene #16701  (Chrivia et al., 1993) |
| pcDNA3 IST-Ty | IST-Ty expression vector | (Huang et al., 2022) |
| pcDNA3 GRA16-Ty | GRA16-Ty expression vector | This paper |
| pcDNA3 GRA24-Ty | GRA24-Ty expression vector | This paper |
| pcDNA3 GRA24 ∆R1/∆R2 -Ty | GRA24∆R1/∆R2 -Ty expression vector | This paper |
| pcDNA3 GRA28-Ty | GRA28-Ty expression vector | This paper |
| 5×-GAS-Firefly luciferase | GAS response element driving Firefly luciferase | Gift from Andrew Bowie  (Lu et al., 2019) |
| pRL-TK | Mammalian co-reporter vector for the weak constitutive expression of wild-type Renilla luciferase | Promega cat# E2231 |

**References:**

Askew, E.B., Bai, S., Blackwelder, A.J., and Wilson, E.M. (2010). Transcriptional synergy between melanoma antigen gene protein-A11 (MAGE-11) and p300 in androgen receptor signaling. J Biol Chem *285*, 21824-21836.

Chrivia, J.C., Kwok, R.P., Lamb, N., Hagiwara, M., Montminy, M.R., and Goodman, R.H. (1993). Phosphorylated CREB binds specifically to the nuclear protein CBP. Nature *365*, 855-859.

Etheridge, R.D., Alaganan, A., Tang, K., Lou, H.J., Turk, B.E., and Sibley, L.D. (2014). The Toxoplasma pseudokinase ROP5 forms complexes with ROP18 and ROP17 kinases that synergize to control acute virulence in mice. Cell Host Microbe *15*, 537-550.

Huang, Z., Liu, H., Nix, J., Xu, R., Knoverek, C.R., Bowman, G.R., Amarasinghe, G.K., and Sibley, L.D. (2022). The intrinsically disordered protein TgIST from Toxoplasma gondii inhibits STAT1 signaling by blocking cofactor recruitment. Nat Commun *13*, 4047.

Lu, Y., Stuart, J.H., Talbot-Cooper, C., Agrawal-Singh, S., Huntly, B., Smid, A.I., Snowden, J.S., Dupont, L., and Smith, G.L. (2019). Histone deacetylase 4 promotes type I interferon signaling, restricts DNA viruses, and is degraded via vaccinia virus protein C6. Proc Natl Acad Sci U S A *116*, 11997-12006.

Markus, B.M., Bell, G.W., Lorenzi, H.A., and Lourido, S. (2019). Optimizing Systems for Cas9 Expression in Toxoplasma gondii. mSphere *4*.

Rosenberg, A., and Sibley, L.D. (2021). Toxoplasma gondii secreted effectors co-opt host repressor complexes to inhibit necroptosis. Cell Host Microbe *29*, 1186-1198 e1188.

Shen, B., Brown, K.M., Lee, T.D., and Sibley, L.D. (2014). Efficient gene disruption in diverse strains of Toxoplasma gondii using CRISPR/CAS9. mBio *5*, e01114-01114.
